# Supplementary material for: Three-Dimensional Bioprinting with Alginate by Freeform Reversible Embedding of Suspended Hydrogels with Tunable Physical Properties and Cell Proliferation
Source: Bioengineering (Basel). 2022 Dec 15;9(12):807. doi: 10.3390/bioengineering9120807 (PMC9774270; doi:10.3390/bioengineering9120807)
Supplement: Supplementary file 1 [file bioengineering-09-00807-s001.zip › bioengineering-2015043-supplementary.pdf]

bioengineering

Supplementary Materials

Three-Dimensional Bioprinting with Alginate by Freeform Reversible Embedding of Suspended Hydrogels with Tunable Physical Properties and Cell Proliferation

Yuanjia Zhu [1,2], Charles J. Stark [1], Sarah Madira [1], Sidarth Ethiraj [1], Akshay Venkatesh [1], Shreya Anilkumar [1], Jinsuh Jung [1], Seunghyun Lee [1], Catherine A. Wu [1], Sabrina K. Walsh [1], Gabriel A. Stankovich [1] and Yi-Ping Joseph Woo [1,2,*]

[1] Department of Cardiothoracic Surgery, Stanford University, Stanford, CA 94305, USA
[2] Department of Bioengineering, Stanford University, Stanford, CA 94305, USA
* Correspondence: joswoo@stanford.edu

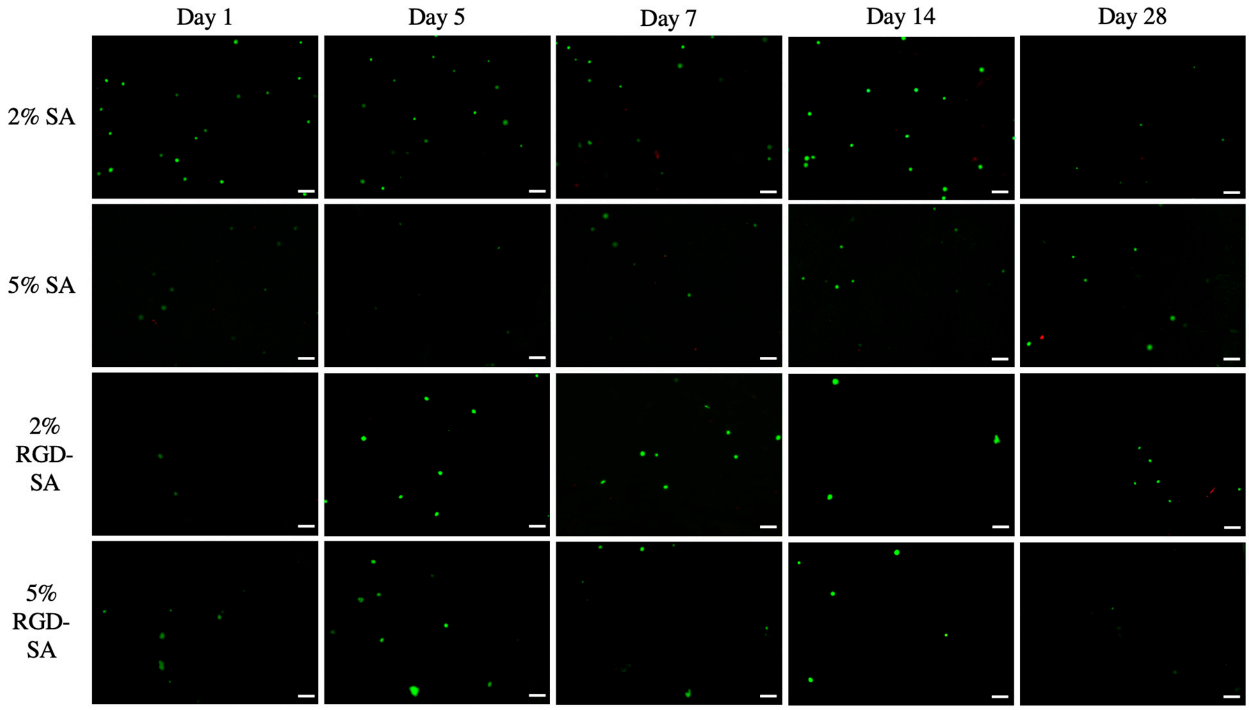

Figure S1. Exemplary live dead images of adult human dermal fibroblasts in different alginate bio-inks after 3D printing over 28 days. Scale bar = 100 µm. SA: sodium alginate; RGD-SA: RGD-modified sodium alginate.

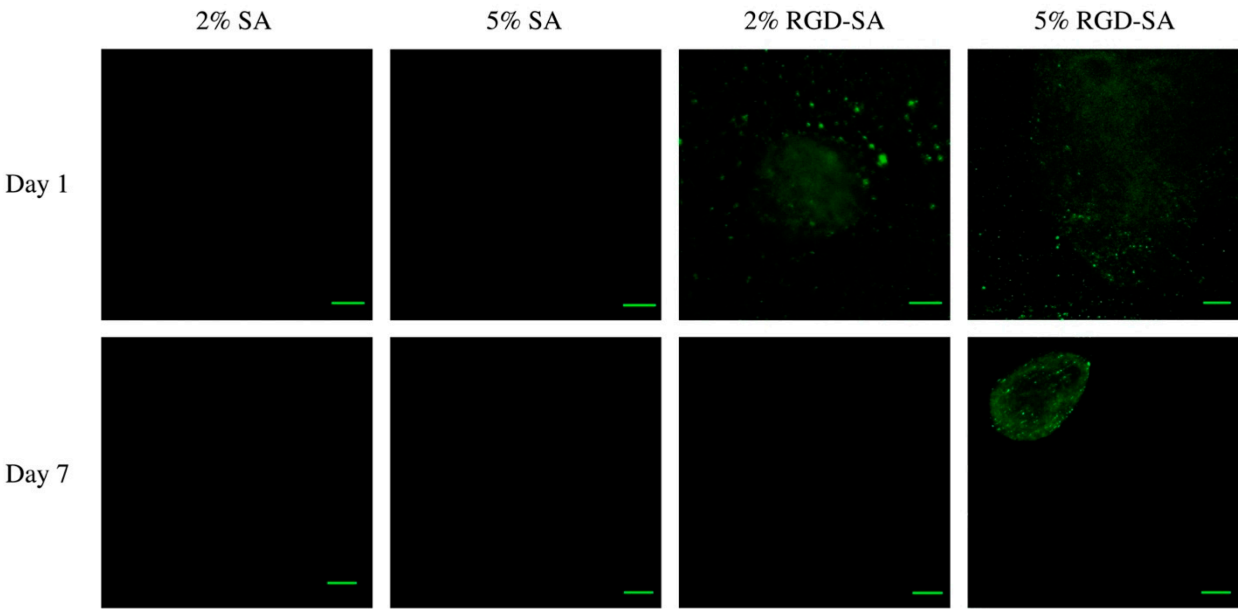

Figure S2. Integrin signals of cells printed in alginates 1 and 7 days after bioprinting. Integrin was present in both RGD-modified alginates 1 day after bioprinting, but after 7 days of culturing, only 5% RGD-SA demonstrated enhanced integrin signals with clustering. SA: sodium alginate. Scale bar = 5 μm.

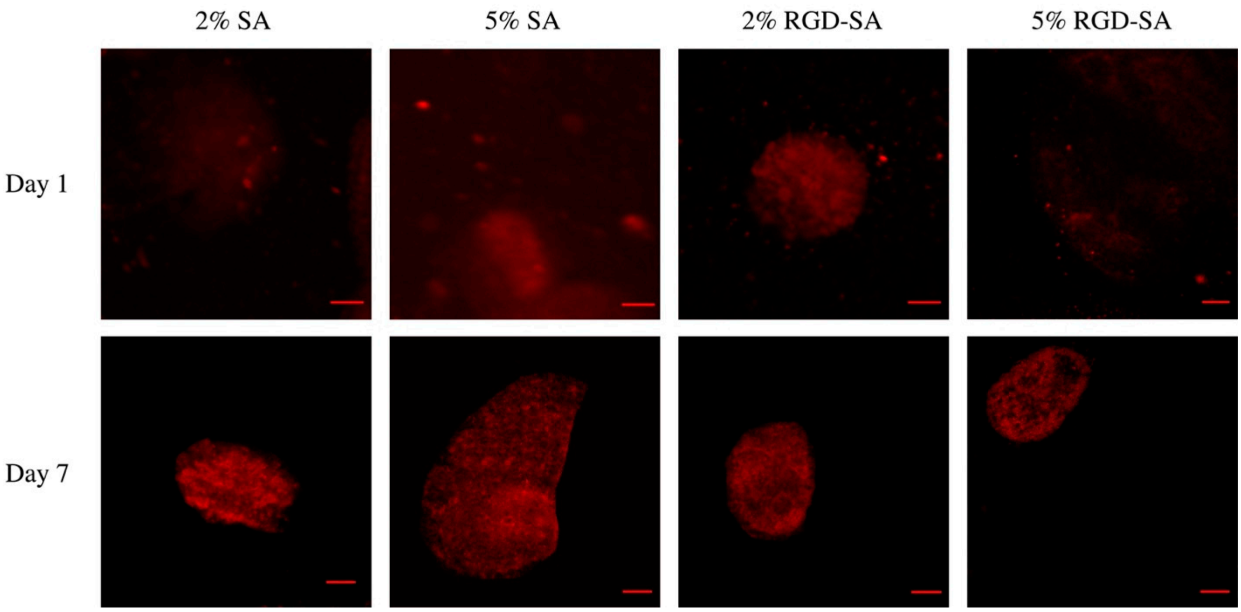

Figure S3. Actin signals of cells printed in alginates 1 and 7 days after bioprinting. Actin signals were well visualized in different alginates at both 1 and 7 days after bioprinting. More pronounced actin formalization was observed after 7 days compared to 1 day after bioprinting. SA: sodium alginate. RGD-SA: RGD-modified sodium alginate. Scale bar = 5 μm.

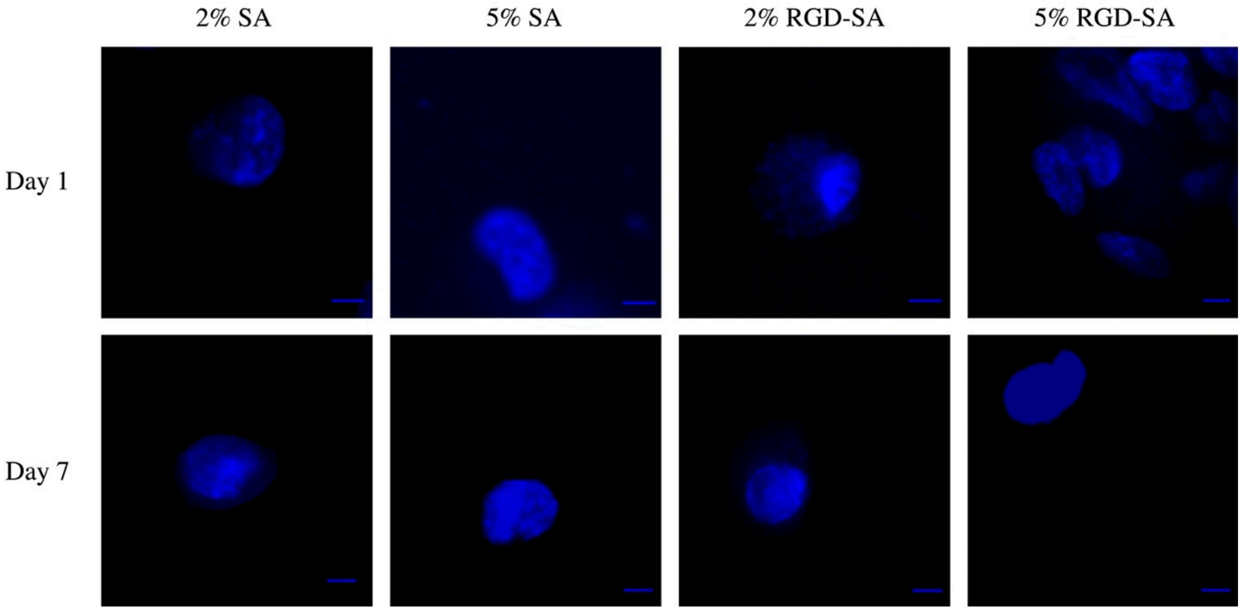

Figure S4. DAPI signals of cells printed in alginates 1 and 7 days after bioprinting. No changes in DAPI signals were observed in different alginates at 1 and 7 days after bioprinting. SA: sodium alginate. RGD-SA: RGD-modified sodium alginate. Scale bar = 5 μm.
